# Retraction: Prevalence of p53 dysregulations in feline oral squamous cell carcinoma and non-neoplastic oral mucosa

**DOI:** 10.1371/journal.pone.0348051

**Published:** 2026-04-27

**Authors:** The PLOS One Editors

After publication of this article [1], concerns were raised regarding Fig 1. Specifically, there appear to be multiple sets of repetitive elements within Fig 1F.

The corresponding author stated that in the original micrograph for Fig 1F (negative control sample), the subepithelial area showed some irregularities originating from tears in the tissue section, and that during figure preparation, the lamina propria was adjusted to improve the visual continuity of the section. The corresponding author provided the original micrograph for Fig 1F which showed significant differences, including extensive tissue tears, compared to Fig 1F in [1], and therefore the *PLOS One* Editors have concerns that this panel may not adequately demonstrate lack of positive staining.

In light of the above concerns which call into question the integrity and reliability of the results in [1], the *PLOS One* Editors retract this article.

ARe, DT, GB, and SS did not agree with the retraction. PDB, LM, JL, ARi, and EB either did not respond directly or could not be reached.

ARe, DT, and SS stand by the article’s findings.
